# The same but different: impact of animal facility sanitary status on a transgenic mouse model of Alzheimer’s disease

**DOI:** 10.1128/mbio.04001-24

**Published:** 2025-04-17

**Authors:** Caroline Ismeurt-Walmsley, Patrizia Giannoni, Florence Servant, Linda-Nora Mekki, Kevin Baranger, Santiago Rivera, Philippe Marin, Benjamin Lelouvier, Sylvie Claeysen

**Affiliations:** 1 IGF, Univ. Montpellier, CNRS, INSERM, Montpellier, Occitanie, France; 2 VAIOMER, Labège, France; 3 Aix-Marseille Univ, CNRS, INP, Inst Neurophysiopathol, Marseille, Provence-Alpes-Côte d'Azur, France; University of California, Davis, Davis, California, USA

**Keywords:** Alzheimer’s disease, gut microbiota, animal facility sanitary status, cognition, amyloid pathology

## Abstract

The gut-brain axis has emerged as a key player in the regulation of brain function and cognitive health. Gut microbiota dysbiosis has been observed in preclinical models of Alzheimer’s disease and patients. Manipulating the composition of the gut microbiota enhances or delays neuropathology and cognitive deficits in mouse models. Accordingly, the health status of the animal facility may strongly influence these outcomes. In the present study, we longitudinally analyzed the fecal microbiota composition and amyloid pathology of 5XFAD mice housed in a specific opportunistic pathogen-free (SOPF) and a conventional facility. The composition of the microbiota of 5XFAD mice after aging in conventional facility showed marked differences compared to WT littermates that were not observed when the mice were bred in SOPF facility. The development of amyloid pathology was also enhanced by conventional housing. We then transplanted fecal microbiota (FMT) from both sources into wild-type (WT) mice and measured memory performance, assessed in the novel object recognition test, in transplanted animals. Mice transplanted with microbiota from conventionally bred 5XFAD mice showed impaired memory performance, whereas FMT from mice housed in SOPF facility did not induce memory deficits in transplanted mice. Finally, 18 weeks of housing SOPF-born animals in a conventional facility resulted in the reappearance of specific microbiota compositions in 5XFAD vs WT mice. In conclusion, these results show a strong impact of housing conditions on microbiota-associated phenotypes and question the relevance of breeding preclinical models in specific pathogen-free (SPF) facilities.

**IMPORTANCE:**

Housing conditions affect the composition of the gut microbiota. Gut microbiota of 6-month-old conventionally bred Alzheimer's mice is dysbiotic. Gut dysbiosis is absent in Alzheimer's mice housed in highly sanitized facilities. Transfer of fecal microbiota from conventionally bred mice affects cognition. Microbiota of mice housed in highly sanitized facilities has no effect on cognition.

## INTRODUCTION

Among the multiple factors involved in the development of sporadic Alzheimer’s disease (AD), the identification of the microbiota-gut-brain axis provided novel insight into one of the underlying mechanisms and its potential to develop therapeutic strategies. Studies aimed at manipulating the gut microbiota of AD mouse models have shown the beneficial effect of such modulations on AD features. Indeed, antibiotic cocktails can prevent the development of amyloid burden and associated neuroinflammation in several commonly used transgenic mouse models of AD, such as APP/PS1, APP/PS1-21 and 5XFAD mice (1–3). Similarly, probiotic treatments prevent memory impairment in amyloid-injected murine models (4, 5).

Modulating the intestinal microbiota can also be achieved by fecal microbiota transplantation (FMT), which allows engrafting microbiota into a recipient’s intestinal tract. FMT has shown promising effects on AD prevention: two case reports indicated a gradual improvement in a patient’s mini-mental state examination (MMSE) score assessing cognitive function, following FMT to treat a *Clostridium difficile* infection (6, 7). Likewise, the microbiota of WT mice prevents cognitive deficits and amyloid markers in APP/PS1 and 5XFAD mice of the same age (8, 9). Moreover, Cryan and colleagues demonstrated that FMT from young to aged mice improves the cognitive performance of the latter (10).

These beneficial effects prompted analyses of the microbiota composition associated with AD. It is now widely accepted that AD patients (11–18), mouse models of amyloidosis (8, 19, 20), or of Tau pathology (21) exhibited dysbiosis of the gut microbiota. The transfer of this dysbiotic fecal microbiota from 5XFAD mice into WT mice induced memory impairments associated with neuroinflammation (19, 20). Altogether, this highlights a causal relationship between microbiota composition and the development of AD-associated phenotypes (22). However, bacteria strains differ from one study to another, which underlines the importance of considering the variables affecting microbiota composition, such as animal sex, age, diet, and analysis method. The sanitary status of animal housing might also have a strong influence, as specific and opportunistic pathogen-free (SOPF) and specific pathogen-free (SPF) housing facilities have been implemented to guarantee user safety because of the absence of numerous zoonotic pathogens, standardize animal health status, and limit variables in experiments, thus facilitating their reproducibility.

In the present study, we investigated the impact of the sanitary status of the animal facility on the fecal microbiota composition and amyloid pathology of 5XFAD mice. We also explore whether the transplantation of this dysbiotic microbiota into WT mice has an impact on cognition, depending on the breeding facility of the donor mice.

## MATERIALS AND METHODS

### Animal facilities

Conventional housed mice were bred in the Faculté de Médecine Secteur Nord, INP, UMR7051 CNRS/AMU, Marseille, France, and SOPF mice in the Plateau Central d'Élevage et d'Archivage, CNRS, Montpellier, France. Micro-organisms excluded in this SOPF animal facility are listed in Table S1. WT recipient mice*, i.e.*, C57Bl/6J were purchased from Janvier-Labs (France).

### Mice

5XFAD mice (23, 24) overexpress human amyloid precursor protein gene (*APP*) bearing Swedish (K670N, M671L), Florida (I716V), and London (V717I) familial AD mutations, and human presenilin one gene (*PS1*) carrying M146L and L286V mutations, both under the control of the neuronal mouse Thy1 promoter. The 5XFAD strain (B6/SJL background) was backcrossed more than 10 generations in C57BL/6 J mice by crossing hemizygous transgenic mice (carrying only one copy of the transgene, designated 5XFAD^Tg^/_0_) with C57BL/6J F1 breeders (Janvier-Labs, Le-Genest-Saint-Isle, France). Litters used in the current study were obtained by crossing hemizygous 5XFAD male with WT females, thus ensuring that pups were born with a naive microbiota. Mice were housed in the respective facilities for more than three generations. At weaning (P21), 5XFAD^Tg^/_0_ and WT littermates were separately caged (three to five animals) to avoid coprophagy between animals of different genotypes. Mice were kept with a 12 h day/night cycle (120–150 LUX) at 21-24°C and 50 ± 10% humidity. Food and water were available *ad libitum* (SAFE A03, A04 SAFE, Augy, France). SOPF water was softened to TH 3.5/4°fH, UV treated, and filtered to 0.2 µM. Tap water was used in conventional housing. In the SOPF facility, mice were housed in ventilated 501 cm² cages (GM500, Techniplast, Decines Charpieu, France) on poplar bedding (SAFE select fine, SAFE, Augy, France). In conventional facilities, mice were housed on similar bedding in 530 cm² cages without filtering lids on standard racks (1284L EUROSTANDARD TYPE II L, Techniplast, Decines Charpieu, France). All animal care and experimental procedures were performed in accordance with National and European regulations (EU directive N°2010/63) and the care guidelines of Montpellier University (authorization B34-417-24, approved protocol #21222).

### Fecal microbiota preparation and administration

The fecal microbiota was sampled at 2 months of age, when amyloid accumulation began but before the onset of cognitive deficits in 5XFAD mice, and at 6 months of age, when the amyloid load was intense and cognitive deficits had set in (24). Following sacrifice of the donor mice (*n* = 4–8, sex-mixed), the fecal contents of the distal and proximal colon were processed within 2 h of sampling. The material from all animals was pooled, weighed, and diluted in sterile PBS at 100 mg/mL. Samples were homogenized in a vortex mixer for 3 min, centrifuged at 800 × *g* for 2 min at 4°C, aliquoted, and stored at −20°C. Each day of treatment, aliquoted fecal material was diluted 1:10 with sterile PBS, and then WT C57Bl/6J mice (sex-mixed) received 100 µL by oral gavage. A sham group of control mice received 100 µL of sterile PBS. Mice were treated twice a week from 8 to 16 weeks of age. Sex may influence the composition of the gut microbiota (25). Despite a half-and-half mixed design, this study was not powered to detect differences between the sexes.

### Novel object recognition test

The novel object recognition (NOR) test (26) was carried out in an open field in a dimly lit room (20–30 Lux). On day 1, mice were habituated to the empty arena for 10 min. On day 2, mice were trained to explore two objects (familiar objects) for 10 min and then returned to their home cage. On day 3, they were subjected to a 5 min restitution session in which one of the two (familiar) objects was replaced by a new one, i.e., novel object. All sessions were video recorded, and analyses were blindly performed to quantify the time the mouse actively explored each object. The discrimination index corresponding to [(exploration time of novel object – exploration time of familiar object)/total exploration time] was then calculated.

### Amyloid load analysis

Thirty-five μicro meter coronal sections of mouse brains were cut on a cryostat and then mounted on slides (SuperFrost Ultra Plus, Epredia, France) in Tris-Gelatin and dried at room temperature (RT) for 24 h. The following day, slides were washed in deionized H_2_O (H_2_Od), dehydrated, and then incubated in a 1:1 mixture of 96% EtOH (CarloErba, France) and Chloroform (HoneyWell, Germany) for 30 min at RT. The slides were then rehydrated, incubated in 0.1% Thioflavin S (Sigma-Aldrich, France) for 10 min, and washed in 70% EtOH for 5 min and then in H_2_Od. The nuclei were stained with DAPI before the slides were coverslipped (Marienfeld, Germany) with mounting medium (Pertex, Histolab, Sweden). Mosaic images were acquired with an upright fluorescence microscope (Leica Thunder, sCMOS Leica DFC9000 camera) with a 10× objective (Plan Aprochromat 0.45) and the following parameters: Hoechst, emission filter centered at 407 nm and exposure time of 4 ms; GFP: emission filter centered at 525 nm and exposure time of 60 ms. For each section, the whole hippocampus and parietal cortex were blindly examined to determine the number and the total area of amyloid deposits for each region. Two to three sections per animal were analyzed.

### 16S rRNA targeted metagenomic analysis

Individual feces were collected from donor mice, frozen in liquid nitrogen, and stored at −80°C until use. The bacterial population present in the samples was determined by targeted sequencing of the V3-V4 variable regions of the 16S rRNA gene according to a protocol established by Vaiomer, France. The workflow classification is restricted to bacteria. The extraction steps, library construction, sequencing, and bioinformatics pipelines have been previously described (27, 28). The targeted microbiome sequences were analyzed using the bioinformatics pipeline (including *LEfSe*—Linear discriminant analysis Effect Size) established by Vaiomer based on the Find Rapidly OTUs with Galaxy Solution (FROGS) guidelines (28, 29). The 16S rRNA gene sequencing data presented in the study are deposited in the ENA repository, accession number PRJEB86885.

### Statistical analysis

The impact of FMT on object time exploration was determined by ANOVA followed by post-hoc test (Bonferroni’s), after verification of Gaussian distribution (Shapiro–Wilk normality test) and homogeneity of sample variance (Brown-Forsythe’s and Bartlett’s tests). For discrimination indexes, Student’s *t*-test was used. For all statistical tests, a *P* < 0.05 was considered significant. Analyses were performed using Prism 9 (GraphPad, MA, USA). For metagenomic analyses, the Mann-Whitney test was used when comparing two groups, and the Kruskal-Wallis test for comparisons involving more than two groups.

## RESULTS

### Dysbiotic fecal microbiota in 6-month-old, conventionally bred 5XFAD mice

**Fig 1:**
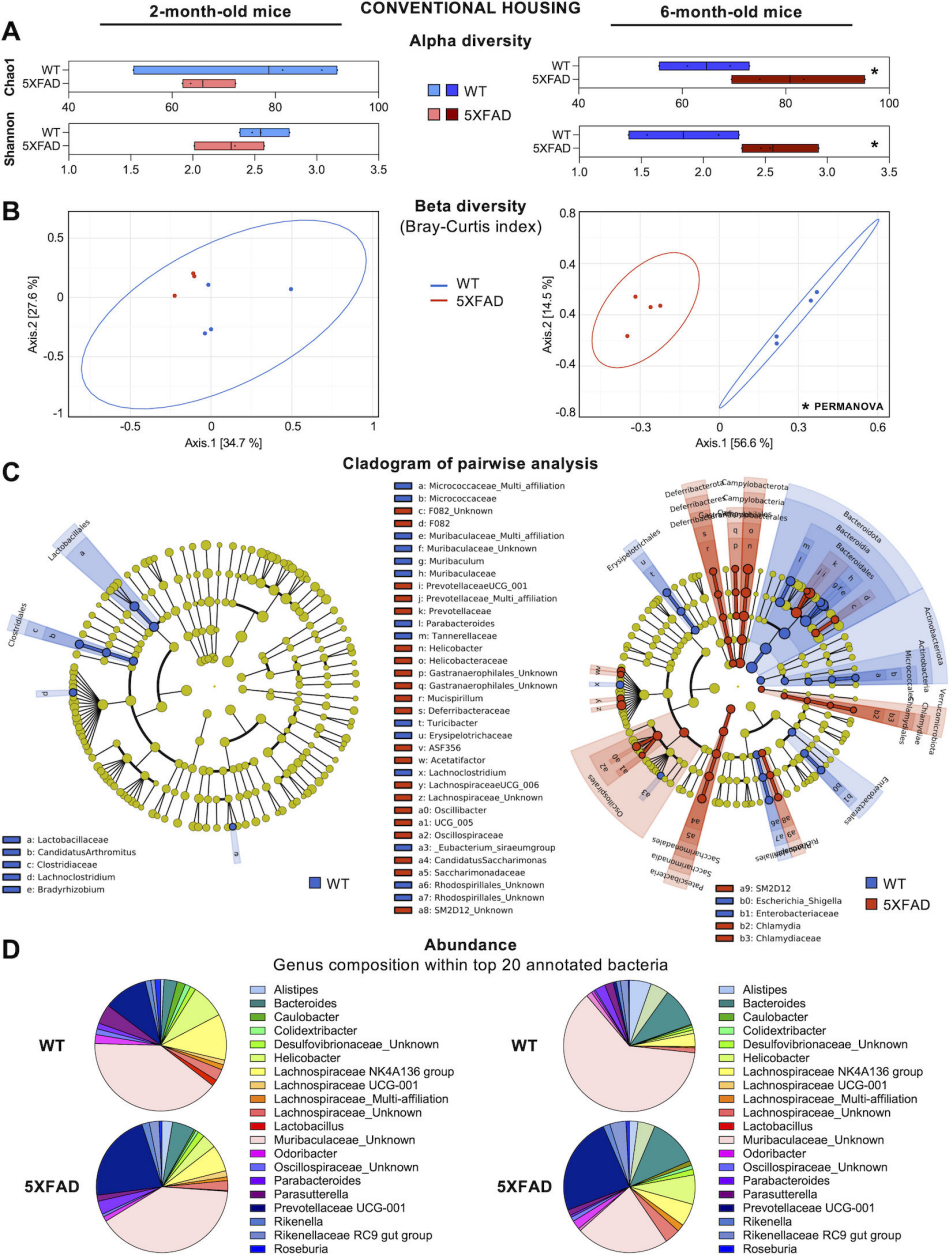
Fecal microbiota composition of 5XFAD and WT littermates in conventional housing. (A) Alpha diversity indexes at the genus level. Data are presented as minimum to maximum and mean. Each dot represents a mouse (*n* = 3–4). *P < 0.05 vs WT (Kruskal-Wallis). (**B**) Beta diversity was assessed by principal coordinates analysis using the Bray-Curtis index. *P < 0.05 vs WT (PERMANOVA). (**C**) Cladogram showing the significant increase in OTU and bacterial taxa identified by LEfSe analysis. In blue: more abundant in WT; in red: more abundant in 5XFAD. (**D**) Synthetic diagram presenting the relative abundance of top 20 annotated genera. Left panels: data from 2-month-old mice. Right panels: data from 6-month-old mice.

To investigate the impact of sanitary status on the composition of the microbiota of 5XFAD mice, we first conducted 16S rRNA-targeted metagenomic analysis of fecal samples collected from 2- and 6-month-old mice bred in conventional housing. At 2 months of age, 5XFAD mice showed a trend toward decreased alpha diversity for all indexes, compared to WT littermates (Fig. 1A; Fig. S1A). The Chao1 index reflects the richness of the samples, while the Shannon and Simpson indexes reflect both the richness and evenness of the diversity of samples (Shannon being more sensitive to evenness and Simpson to richness). Beta diversity of each group, illustrated by Principal Coordinate Analysis (PCoA) of the Bray-Curtis index (comparing dissimilarity between samples based on species abundance), showed no significant difference (PERMANOVA & PERMDISP) between the bacterial composition of 5XFAD and WT mice (Fig. 1B; Fig. S1A). Statistical pairwise comparison using LEfSe analysis revealed only a few significantly different bacterial phylotypes compared to WT littermates (Fig. 1C), as did the relative abundance of bacterial genera (Fig. 1D).

At 6 months of age, 5XFAD mice showed a significant increase in alpha diversity for the Chao1 (*P* = 0.04), Shannon (*P* = 0.02), and Simpson (*P* = 0.02) indexes (Fig. 1A; Fig. S1B), compared to WT mice. Beta diversity revealed significant dissimilarity of bacterial populations in the two genotypes in both Bray-Curtis (Fig. 1B, PERMANOVA *P* = 0.02) and Unifrac indexes (Fig. S1B, PERMANOVA *P* = 0.03, PERMDISP *P* = 0.03). Statistical pairwise comparison and relative abundance of bacterial genera (Fig. 1C and D) confirmed that 6-month-old 5XFAD mice had a marked different bacterial composition, compared to WT littermates of the same age, presenting a global reduced proportion of Bacteroidota phylum which has been documented by others (30).

Globally, the relative abundance of the top 20 genera within the annotated bacteria (Fig. 1D) showed that an unknown genus belonging to the *Muribaculaceae* family was the most abundant (40% of top 20 genera in WT and 5XFAD 2-month-old mice; increasing to 62% in WT 6-month-old mice; and decreasing to 23% in 5XFAD 6-month-old mice). We also found a 2- and 31-fold increase in *Prevotellaceae UCG-001*, the second most abundant genus, in 5XFAD mice compared to WT mice at 2 and 6 months, respectively. Of note, we also detected in all groups the clear presence of *Helicobacter* genus (0.8%–8%), a bacterial genus that is excluded from animal facilities with restricted sanitary status (Table S1). In other studies, significant decreases in *Muribaculum* genus or *Muribaculaceae* family were similarly observed in 6- to 8-month-old 5XFAD mice, which also showed an increase in the *Helicobacter* genus, suggesting that they were probably housed in conventional facilities (30, 31).

Taken together, these metagenomic analyses showed that 5XFAD mice in conventional housing develop a dysbiotic fecal microbiota with age, compared to their WT littermates.

### Absence of dysbiosis of fecal microbiota in 6-month-old, SOPF-bred 5XFAD mice

**Fig 2:**
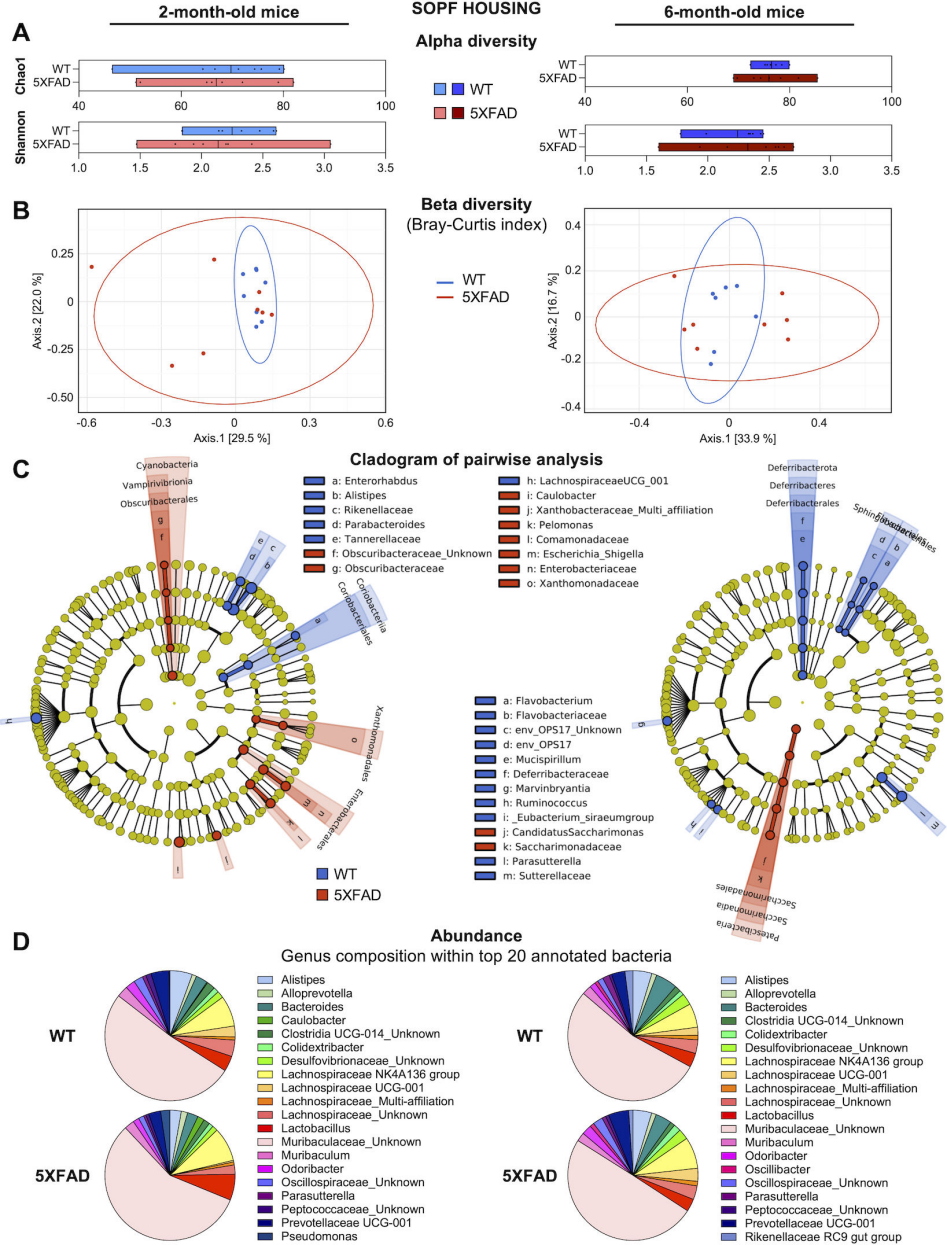
Fecal microbiota composition of 5XFAD and WT littermates in SOPF housing. (A) Alpha diversity indexes at the genus level. Data are presented as minimum to maximum and mean. Each dot represents a mouse (*n* = 7–8). (**B**) Beta diversity was assessed by principal coordinates analysis using the Bray-Curtis index. (**C**) Cladogram showing the significant increase in OTU and bacterial taxa identified by LEfSe analysis. In blue: more abundant in WT; in red: more abundant in 5XFAD. (**D**) Synthetic diagram presenting relative abundance of the top 20 annotated genera. SOPF, specific and opportunistic pathogen-free; left panels, data from 2-month-old mice; right panels, data from 6-month-old mice.

We then carried out 16S rRNA-targeted metagenomic analysis of fecal samples from 5XFAD mice bred in SOPF facility to monitor the development of dysbiosis between the ages of 2 and 6 months. At both ages, we found no significant difference in either alpha diversity (using Chao1, Shannon, and Simpson indexes) or beta diversity (using the Bray-Curtis and Unifrac indexes) between WT and 5XFAD mice (Fig. 2A and B; Fig. S1B). At 6 months of age, the bacterial composition was still highly similar in both groups (Fig. 2B). Statistical pairwise comparison and relative abundance of taxa at genus level confirmed no major divergence between the two phenotypes (Fig. 2C and D). In the SOPF facility, an unknown genus belonging to the *Muribaculaceae* family was still the most abundant among the top 20 annotated genera (Fig. 2D). We observed no change in the relative abundance of *Prevotellaceae UCG-001* genus in 6-month-old 5XFAD mice compared to WT mice (3% vs. 5%).

Together, these analyses indicate that a SOPF environment prevents the development of a dysbiotic fecal microbiota in 6-month-old 5XFAD mice, compared to WT littermates.

### Impact of donor housing on memory alteration by chronic transplantation of fecal microbiota

**Fig 3:**
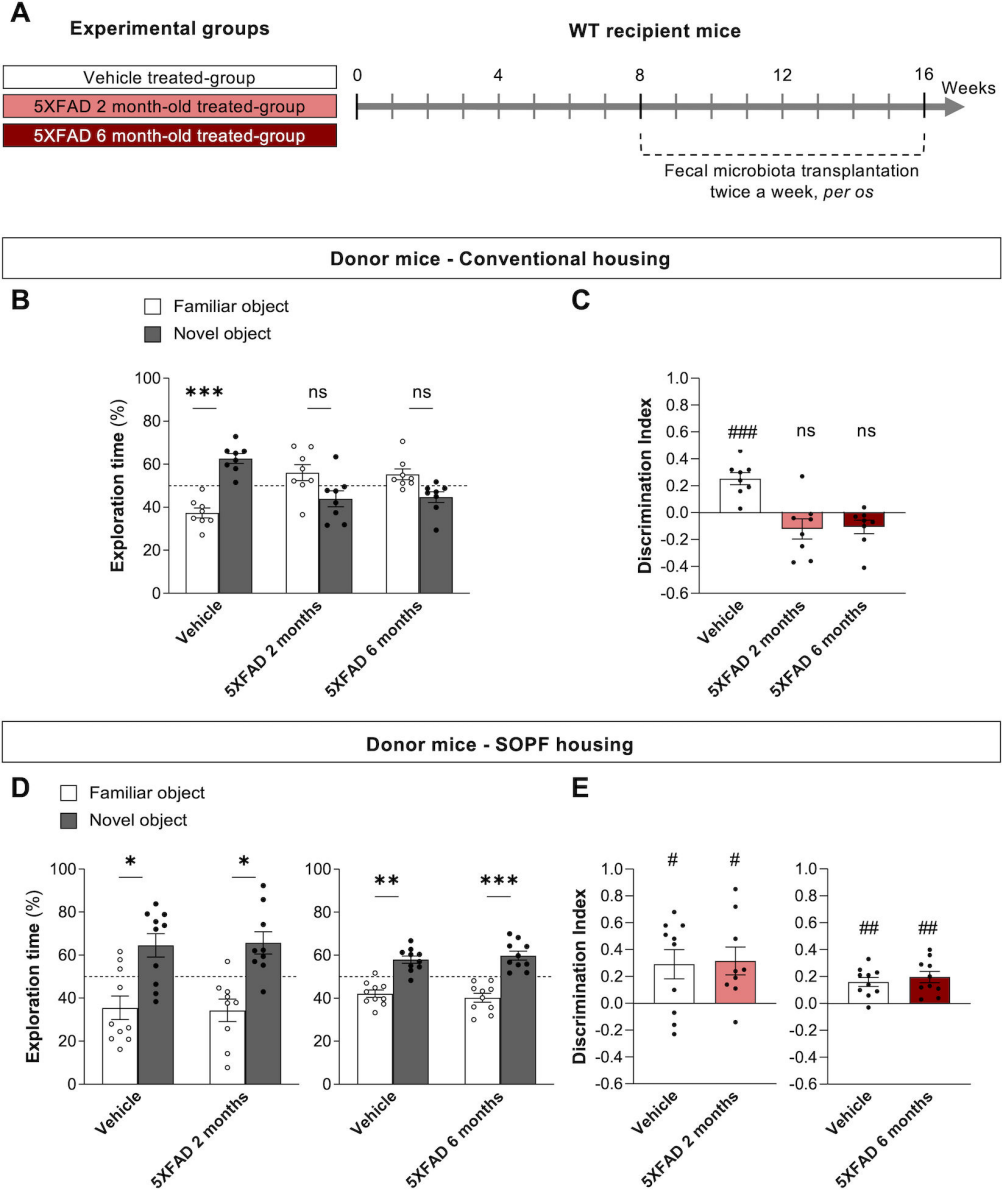
Impact of donor housing conditions on cognitive performance alteration induced by FMT. (A) Experimental design. (**B–E**) Novel object recognition test in 16-week-old mice performed with a 24 h intersession interval. (**B, D**) Time spent by mice exploring familiar and novel objects during test session. ***P < 0.001, **P < 0.01, *P < 0.05, significantly different from familiar object (two-way repeated-measures ANOVA, followed by Bonferroni’s test). (**C, E**) Discrimination index calculated by using exploration times in the test session and formula [(novel − familiar)/(familiar + novel)]. Indices positively different from zero: ##*P* < 0.01, #*P* < 0.05 (one sample Student’s *t* test). Data are presented as means ± SEM. Each dot represents a mouse (*n* = 8–10). As a result of two distinct experiments, the vehicle groups are presented separately in D and E panels. SOPF, specific and opportunistic pathogen-free.

To determine whether these distinct microbial compositions have a differential impact on cognition following FMT to WT mice, fecal microbiota from 2- or 6-month-old 5XFAD mice bred in either a conventional or SOPF environment were administered to WT mice by chronic gavage for 2 months (Fig. 3A). The cognitive performance of the recipient mice was assessed at 16 weeks of age using the NOR test. Of note, experiments with conventionally reared animals at 2 and 6 months of age were performed together, thus having the same control group (vehicle-treated mice), whereas experiments with SOPF-bred animals were performed in two batches, thus having independent control groups.

WT recipient mice that received vehicle solution presented normal memory performance, as shown by the higher percentage of exploration time spent exploring the novel object (Fig. 3B and D) and the positive discrimination indexes (Fig. 3C and E). The transplantation of fecal microbiota from conventionally housed 5XFAD mice aged 2 or 6 months induced cognitive deficits in the recipient mice (Fig. 3B and C). In contrast, mice receiving microbiota from 2- or 6-month-old 5XFAD mice bred in SOPF facility showed similar memory performance to that of mice transplanted with the vehicle, as shown by the significant difference in the percentage of exploration time and discrimination indexes (Fig. 3D and E). Collectively, these results demonstrate that the fecal microbiota of 5XFAD mice differentially impacts the cognition of WT recipient mice in the NOR test, depending on the initial housing in conventional vs SOPF facility.

### Re-establishment of a dysbiotic microbiome in SOPF-born 5XFAD mice after 18 weeks in a conventional environment

**Fig 4:**
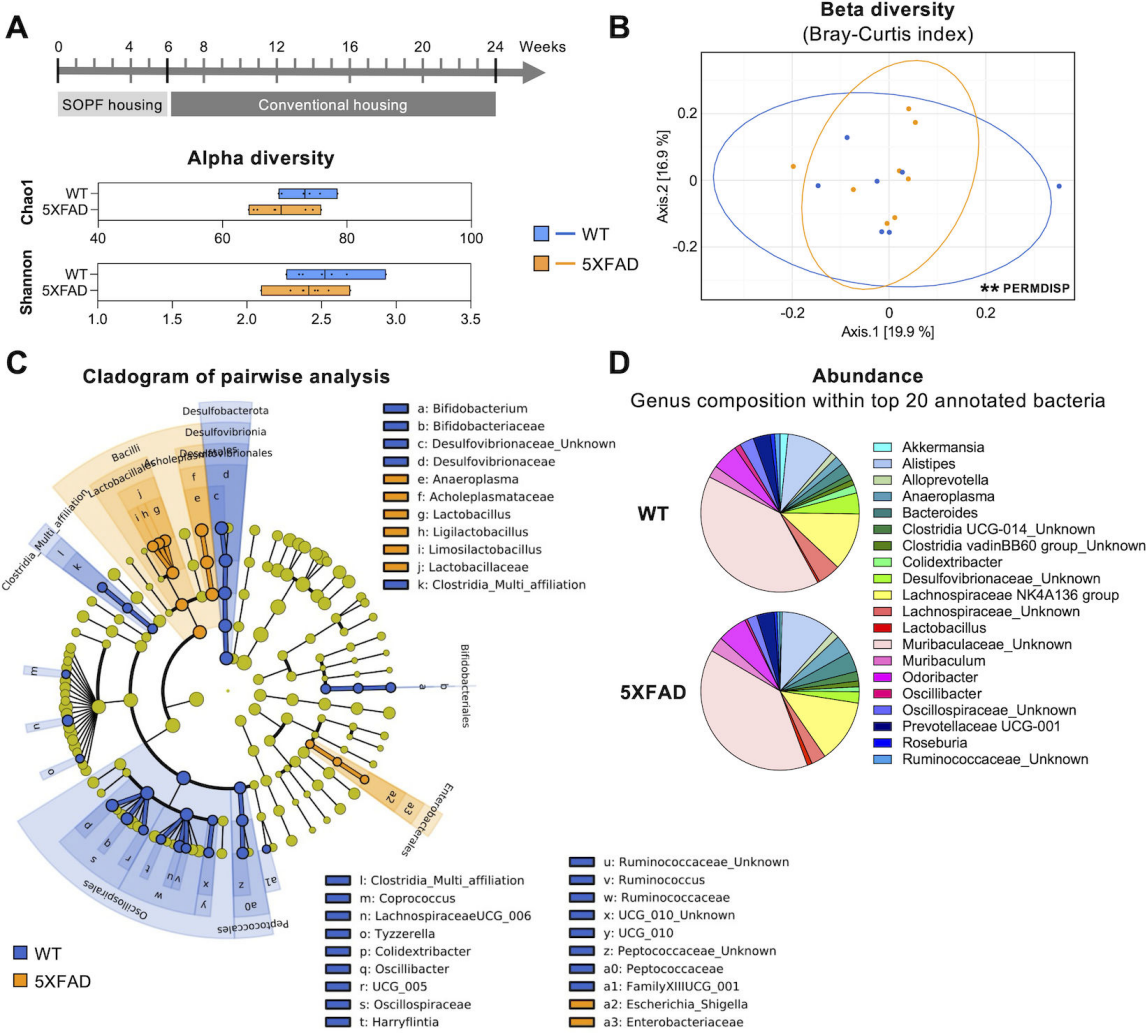
Fecal microbiota composition of SOPF-born 5XFAD and WT littermates after 18 weeks in conventional housing. (A) Alpha diversity indexes at the genus level. Data are presented as minimum to maximum and mean. Each dot represents a mouse (*n* = 7–8). (**B**) Beta diversity was assessed by principal coordinates analysis using the Bray-Curtis index. *P < 0.05 vs WT (PERMDISP). (**C**) Cladogram showing the significant increase in OTU and bacterial taxa identified by LEfSe analysis. In blue: more abundant in WT; in orange: more abundant in 5XFAD. (**D**) Synthetic diagram showing the relative abundance of the top 20 annotated genera.

As co-housing SPF-born mice with conventionally-born mice in a conventional facility significantly increased their gut microbiota diversity (32), we investigated whether SOPF-born 5XFAD mice developed a dysbiosis of fecal microbiota compared to WT littermates after 18 weeks of housing in a conventional environment. Bacterial composition of fecal samples showed no significant modification in alpha diversity for 5XFAD mice after 18 weeks in a conventional environment, compared to WT mice (Fig. 4A), whereas Bray-Curtis dissimilarity and Unifrac distances revealed significant differences in the two groups (PERMDISP, *P* = 0.0099 and *P* = 0.02; PERMANOVA, *P* = 0.1 and *P* = 0.02, respectively, Fig. 4B; Fig. S2). The relative abundance at the genus level and pairwise statistical comparisons showed that WT and 5XFAD mice are differentially colonized by bacteria living in a conventional environment (Fig. 4C and D) and that the composition of their respective microbiota shows greater relative differences than in the SOPF housing (Fig. 2C and D). Taken together, these results demonstrate that rearing SOPF-born animals in conventional housing allows the development of a dysbiotic fecal microbiota in 6-month-old 5XFAD mice.

### Reduction of amyloid pathology in 6-month-old SOPF-bred 5XFAD mice compared to conventionally bred 5XFAD mice

**Fig 5:**
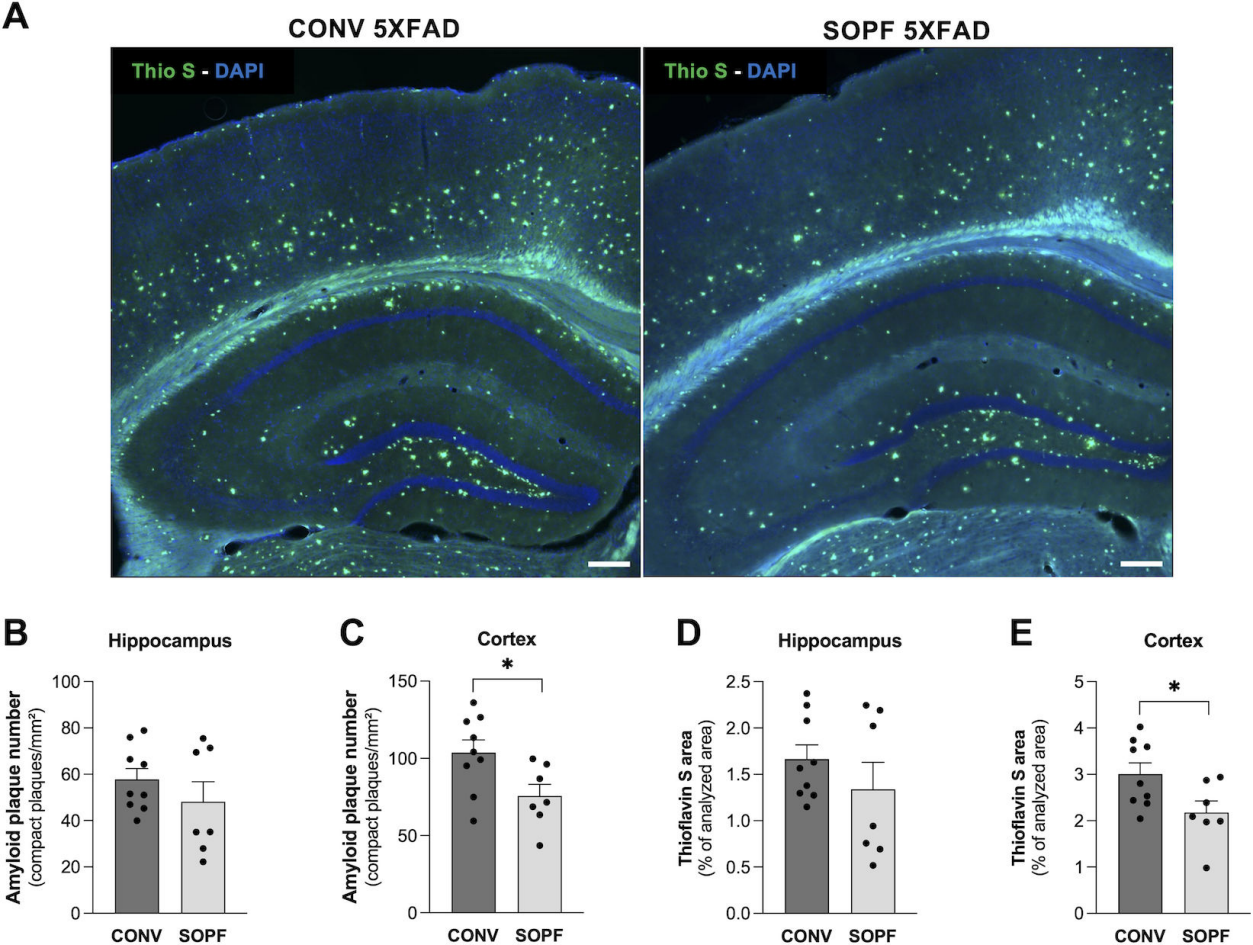
Amyloid load in 6-month-old 5XFAD mice bred conventionally and in SOPF housing. (**A**) Representative images of compact amyloid plaques stained with Thioflavin S (green) and nuclei stained with DAPI (blue). Scale bar: 200 µm, magnification 10×. (**B–E**) Quantification of the number (**B, C**) and area (**D, E**) of amyloid aggregates normalized to the total area of the hippocampus or cortex. *P < 0.05 (Unpaired Student’s *t* test). Data are presented as means ± SEMs. Each dot represents one slice (*n* = 7–9). CONV, conventional; SOPF, specific and opportunistic pathogen-free.

Since germ-free AD mouse models are known to produce less Aβ peptides and deposit in the brain compared to conventionally bred mice (33), we investigated whether 6-month-old 5XFAD mice bred in the SOPF housing exhibit lower brain amyloid loads compared to 5XFAD mice of the same age bred in the conventional facility. Histopathologic staining of compact amyloid deposits with Thioflavin S was performed on hippocampal and cortical sections (Fig. 5A through E). The number of amyloid deposits was significantly reduced in the cortex of SOPF-5XFAD mice compared to conventional 5XFAD mice (−19%, *P* = 0.025, Fig. 5C). Similarly, a significant decrease in the total area of cortical thioflavin staining was observed in the brains of SOPF-5XFAD mice (−18%, *P* = 0.033, Fig. 5E). In the hippocampus, no significant effect was observed for any parameter although half of the animals showed a downward trend (Fig. 5B and D).

## DISCUSSION

**Fig 6:**
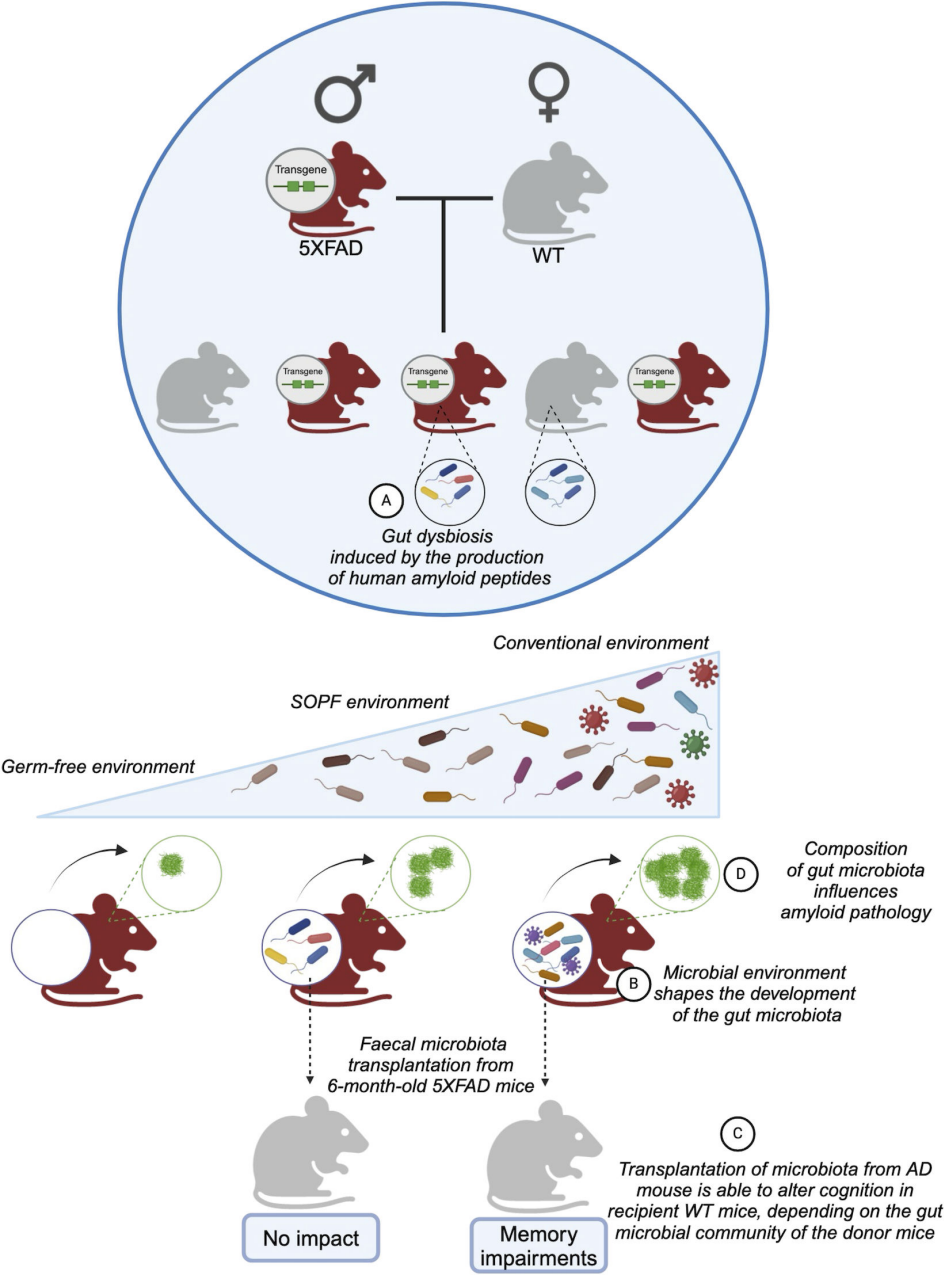
Study summary. (**A**) The transgene in 5XFAD mice induces the development of dysbiotic fecal microbiota compared to WT littermates. (**B**) Environmental microbial diversity influences the development of gut microbiota composition. (**C**) The effect of fecal microbiota transplantation from 6-month-old 5XFAD mice on cognition in WT mice depends on the environment and the donor microbiota. (**D**) Gut microbiota has an impact on amyloid pathology in 5XFAD mice. SOPF, Specific and opportunistic pathogen-free.

In this study, we investigated the impact of the sanitary status of the animal facility on the composition of the fecal microbiota of 5XFAD mice, and whether the transplantation of their dysbiotic microbiota into naive mice affected cognitive performance, depending on the breeding facility of the donor mice (Fig. 6).

The importance of gut microbiota in brain physiopathology is raising an increasing interest, especially in AD (34). Several studies have reported intestinal dysbiosis in AD patients and transgenic mouse models of AD (8, 11, 15, 18–20). Dysbiosis of the gut microbiota can result from well-described external factors, such as the mode of delivery, diet, physical activity, or antibiotic use (35–38). Children inherit part of their mother’s microbiome (39), and the first events that might influence the development of a dysbiotic microbiota occur before birth during *in utero* development. Hsiao and colleagues have shown that maternal immune activation leads to alterations in the composition of the gut microbiota in the offspring (40). In addition, mood disorders, such as anxiety and stress during pregnancy, result in the development of a dysbiotic microbiota in newborn mice (41). In the context of AD, there is a reciprocal relationship between the gut microbiota dysbiosis and the pathology. Modulation of the microbiota by antibiotic treatment reduces the amyloid load in the brains of treated AD mice (2, 42) and, in a mirror image, the injection of Aβ into the lateral ventricle of mice is able to induce an alteration of the gut microbiota 4 weeks later (43). Alterations of the patient lifestyle by AD, such as circadian cycle disruption, changes in eating habits, and physical inactivity, have been shown to induce gut microbiota dysbiosis (44–46). Transplantation of microbiota from AD patients into rats induces memory impairment that correlates with the clinical scores of the donors (47).

In the present work, we confirmed that 5XFAD mice display age-related changes in fecal bacterial composition compared to WT littermates. It should be noted that all matings were performed by pairing 5XFAD males with WT females. Therefore, all newborns were derived from naive mice, meaning that the only difference between 5XFAD mice at birth was the presence of the *APP* and *PS1* transgenes, suggesting that the production of human amyloid peptides and their downstream effects could be responsible for the development of microbial dysbiosis. Confirming this hypothesis, a recent study showed that the knock-in AD mouse models, APP^NL-F^ and APP^NL-G-F^, expressing mutant human APP at physiological levels, exhibit an altered gut microbiota compared to controls (48). Our mating strategy should have avoided any confounding effects due to the maternal microbiome. However, it has recently been shown that paternal gut microbiota can also alter the offspring phenotypes (49). As the mice used here were housed in the respective facilities for more than three generations, we should have fixed the paternal effect associated with the breeding facility.

As maintaining the health status of animal models is a major concern, SOPF facilities have been implemented. We observed that in such a controlled environment, 5XFAD mice developed differences in fecal bacterial composition compared to WT littermates but did not develop dysbiosis at 6 months of age, as observed in conventional housing. These results demonstrate that an environment with restricted microbial diversity can prevent the development of a known phenotype. However, breeding SOPF-born 5XFAD mice in a conventional facility restored the dysbiosis. This finding is supported by data showing that co-housing conventionally raised animals with SPF animals significantly increases the diversity of the latter’s gut microbiota (32).

We then assessed the impact of fecal microbiota transplantation from 5XFAD mice on cognition in naive WT mice. Importantly, transplantation was performed without depletion of the endogenous microbiota, but repeatedly over a 2-month period to mimic a slow and chronic deregulation of the microbiota and to avoid the use of antibiotics that would interfere with the composition of the microbiota. When donor mice are bred in a conventional environment, we observed that the dysbiotic microbiota from 2- and 6-month-old 5XFAD mice induces cognitive impairment in WT mice, while the microbiota of age-matched SOPF-bred 5XFAD mice did not impair memory in recipient mice. As the fecal microbiota of conventionally bred 5XFAD mice is not very different from that of WT mice, we hypothesize that the negative effect on memory of naïve recipients may be due to differences in the fecal metabolic profile, which was not studied here (50, 51). In SOPF-bred 5XFAD mice, the absence of a dysbiotic profile delays the phenotype of the model and might explain the lack of effect of microbiota transfer on cognitive performance. Similar experiments evaluating the cognition in WT mice receiving fecal microbiota from 5XFAD mice purchased from the Jackson Laboratory (Bar Harbor, ME, USA), a procedure reminiscent of our second set of experiments performed with SOPF-bred donors, showed that the FMT from 9-month-old mice to recipient mice depleted of the endogenous microbiota with an antibiotic cocktail leads to deficits in spatial learning and memory in the Morris water maze test in these mice (22). These experiments are difficult to compare with the present study, as experimental paradigms are different and microbiota composition was assessed after or before FMT (in our case, no samples were taken post-FMT). However, a striking difference lies in the amount of *Prevotella* genus, which was found to be reduced in mice subjected to 5XFAD-FMT, compared to WT-FMT in the study of Kim et al*.* (22). In contrast, we found an increase in *Prevotella* in 5XFAD compared to WT, as described by others (52). The housing environment of the recipient mice is also an important factor, as obesity studies have demonstrated that transplantation of human microbiota partially recapitulates obese phenotypes in conventionally reared mice, in contrast to SPF-bred mice, which show no change (53).

The gut microbiota composition also correlates with amyloid pathology in preclinical models and patients (11, 18). Harach et al. showed that amyloid levels were lower when AD mice were bred in germ-free facility than in a conventional environment and that colonization of axenic AD mice with microbiota from conventionally bred mice were able to increase brain amyloid pathology (33). Similarly, in our study, we observed that 6-month-old SOPF-bred 5XFAD mice showed a decrease in amyloid load in the hippocampus and cortex compared to conventionally bred mice of the same age. In transgenic mouse models of amyloidosis, cognitive impairment arises as a result of amyloid production, and slowing amyloidosis can postpone the onset of cognitive deficits (54–56). We could postulate that a more impoverished microbiota composition may be associated with a less pronounced cognitive phenotype in animals, following this classification: GF < SOPF < SPF < Conventional < Real life. In 5XFAD females born and reared in the SOPF, we observed a delay of approximately one month in the onset of cognitive deficits compared to animals from SOPF facilities and a delay in amyloid plaque accumulation (data not shown). However, this must be clearly demonstrated, considering the effect of sex (cognitive deficits appear later in males than in females) and the age of the animals. Later in life, when amyloid reaches a plateau (24, 57), cognitive performance may be similar in animals of different origins. This hypothesis could be extended to other transgenic animal models of amyloidosis, such as APP/PS1 and 3xTg-AD mice, in which such a link between neuropathology and gut microbiota composition has been reported (42, 58, 59). This could also be extended to senescence-accelerated mice (SAM), where an early study reported the absence of amyloid deposition in animals bred in SPF conditions compared to those bred in conventional housing (60). Similarly, in a landmark paper, the germ-free αSyn (ASO) overexpressing mouse model of Parkinson’s disease showed reduced motor deficits compared to SPF ASO mice (61).

This study focused on behavioral studies and metagenomic analysis and did not investigate the mechanisms underlying our observations. In AD, there is a general alteration in metabolism, with changes in short-chain fatty acid (SCFA) (62) and bile acid (63) levels, as well as dysregulation of glucose metabolism and cerebral insulin resistance (64), which have been implicated in cognitive decline and amyloid accumulation (65). In our studies, modulation of the gut microbiota may have influenced these pathways. To better understand whether microbiota-induced changes extend beyond the gut-brain axis and contribute to metabolic dysfunction, additional analyses would be insightful. These include quantifying key SCFAs like butyrate, profiling bile acids, and assessing blood glucose levels, insulin sensitivity, and insulin signaling in the brain. Specific markers such as insulin receptor activation, IRS-1 phosphorylation, and glucose transporter expression could provide further insights. Immune system dysregulation is a key characteristic of AD, with substantial evidence showing progressive pathological changes in both central and peripheral immune responses. These changes evolve over time and appear to be influenced by the gut microbiota (66, 67). Moreover, the maturation of the immune system is highly dependent on the microbiota, and the FMT of conventional microbiota may influence the immune response of recipient mice born in the SOPF facility of the Janvier laboratories or in SPF animal housing (68). A prospective study would focus on these aspects by quantifying circulating inflammatory mediators (pro- and anti-inflammatory cytokines, immune cells) and analyzing markers of neuroinflammation (activation of microglia and astrocytes), as previously reported (1, 54). It would be important to extend our study to GF mice. GF-5XFAD mice showed reduced amyloid pathology and altered microbiota composition (33). We would expect that FMT in GF-5XFAD mice would not induce cognitive impairment in the WT recipient mice used in our study. On the other hand, since FMT from SPF or conventionally bred mice increased amyloid pathology in GF-5XFAD mice (33), such transfer should induce impaired memory performance in recipient mice. However, GF mice should be used with caution in cognitive studies, as they exhibit neurodevelopmental abnormalities and altered neurotransmitter levels (69–72). Finally, the age of the recipient mice is an important factor to consider. The microbiota of aged mice can impair the cognitive performance of young recipients (73) but has a limited capacity for self-restoration (74). It remains unclear whether FMT from 5XFAD to WT mice would lead to more severe cognitive impairment in aged WT recipients. This question could be better addressed using quantitative tests, such as the Morris water maze or the Barnes maze, rather than the NOR test.

In summary, the sanitary status of the animal facility has a strong impact on AD development and associated behavioral deficits in mouse models. As the environment modifies the microbiota and its impact on diseases, different environments will drive divergent phenotypes in animal models. AD mice housed in highly controlled environments (SPF and SOPF) will have an impoverished microbiota and develop less pronounced disease phenotype than the same mice housed in conventional animal facilities. The integration of these data may lead to reconsider the use of animals from conventional housing or colonized with wild microbiota (75) rather than SPF- and SOPF-status animals in studies investigating the relationship between gut microbiota and neurological disorders, such as AD. The translational value of preclinical studies on these complex pathologies should be enhanced by working with models that more closely mimic the natural microbiota.

## SUPPLEMENTAL MATERIAL

Supplementary material mbio.04001-24-s0001.pdf Fig. S1, Fig. S2, and Table S1.

ASM does not own the copyrights to Supplemental Material that may be linked to, or accessed through, an article. The authors have granted ASM a non-exclusive, world-wide license to publish the Supplemental Material files. Please contact the corresponding author directly for reuse.
