# Supplementary material for: The same but different: impact of animal facility sanitary status on a transgenic mouse model of Alzheimer’s disease
Source: mBio. 2025 Apr 17;16(5):e04001-24. doi: 10.1128/mbio.04001-24 (PMC12077201; doi:10.1128/mbio.04001-24)
Supplement: Supplementary material — Fig. S1, Fig. S2, and Table S1. [file mbio.04001-24-s0001.pdf]

The same but different: impact of animal facility sanitary status on a transgenic mouse model of Alzheimer's disease

Caroline Ismeurt-Walmsley[a,1], Patrizia Giannoni[a,2], Florence Servant[b], Linda-Nora Mekki[a], Kevin Baranger[c,3], Santiago Rivera[c], Philippe Marin[a], Benjamin Lelouvier[b], Sylvie Claeysen[a]

[a] IGF, Univ. Montpellier, CNRS, INSERM, Montpellier, France
[b] VAIOMER, Labège, France
[c] Aix-Marseille Univ, CNRS, INP, Inst Neurophysiopathol, Marseille, France

[1] Present address: IGMM, Université de Montpellier, CNRS, F-34293, Montpellier, France
[2] Present address: Equipe Chrome, EA7352, Université de Nîmes, F-30000 Nîmes, France
[3] Present address: LIENSs UMRi 7266 CNRS/La Rochelle Université, F-17000 La Rochelle, France

Correspondence to:
Sylvie Claeysen
IGF - CNRS UMR5203 - INSERM U1191 - Univ. Montpellier
141 rue de la Cardonille, 34094 Montpellier, France
Email: sylvie.claeysen@igf.cnrs.fr

Supplemental material

- **Supplementary figure 1.** Fecal microbiota composition of 5XFAD and WT littermates in conventional and SOPF housing.
- **Supplementary figure 2.** Fecal microbiota composition of SOPF-born 5XFAD and WT littermates after 18 weeks in a conventional environment.
- **Supplementary Table 1.** Exclusion list of the Plateau Central d'Elevage et d'Archivage, CNRS, Montpellier (SOPF animal facility).

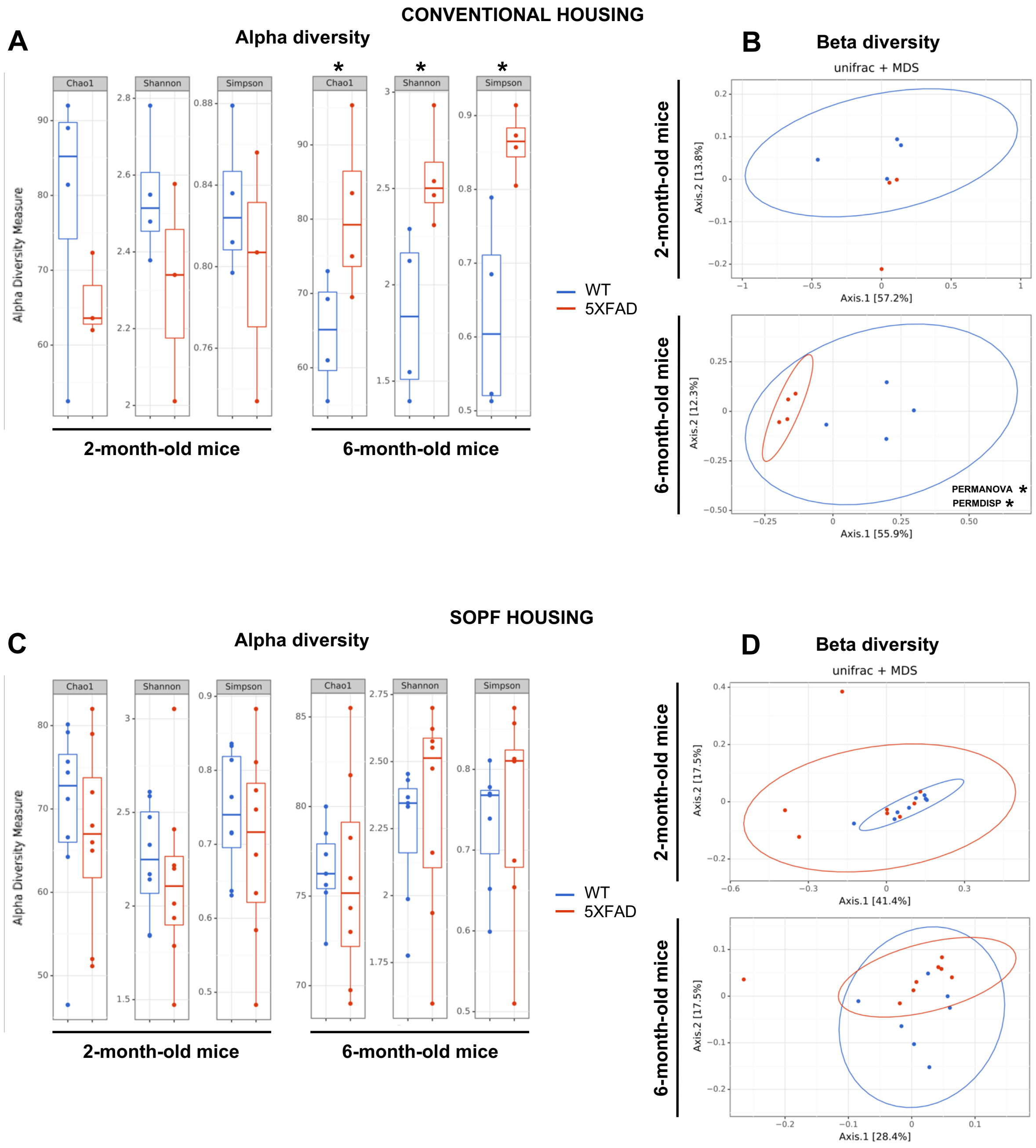

Supplementary figure 1. Fecal microbiota composition of 5XFAD and WT littermates in conventional (A, B) and SOPF (C, D) housing.
(A, C) Alpha diversity indexes at the genus level. Data are presented as 25-75% interquartile range and median. Each dot represents a mouse. *p < 0.05 versus WT (Kruskal-Wallis) **(B, D)** Beta diversity assessed by principal coordinates analysis using Unifrac index. *p < 0.05 versus WT (PERMANOVA or PERMDISP).

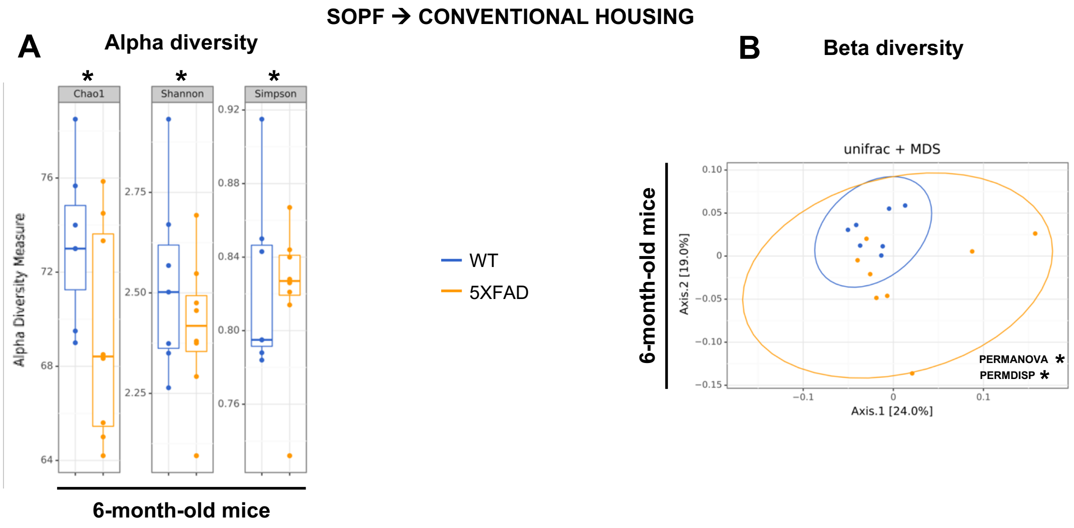

Supplementary figure 2. Fecal microbiota composition of SOPF-born 5XFAD and WT littermates after 18 weeks in a conventional environment.
(A) Alpha diversity indexes at the genus level. Data are presented as 25-75% interquartile range and median. Each dot represents a mouse. *p < 0.05 versus WT (Kruskal-Wallis) **(B)** Beta diversity assessed by principal coordinates analysis using Unifrac index. *p < 0.05 versus WT (PERMANOVA or PERMDISP).

Bacteria	Endo and ectoparasites	Viruses
Bordetella bronchiseptica	*Aspiculuris tetraptera*	Hantavirus
Cilia-associated respiratory bacillus	*Chilomastix*	K virus
Citrobacter rodentium	*Cryptosporidium muris*	Lactate dehydrogenase elevating virus
Clostridium piliforme	*Cryptosporidium parvum*	Lymphocytic choriomeningitis virus
Corynebacterium bovis	*Demodex*	MKPV
Corynebacterium kutscheri	Dermatophytes	Mouse adenovirus type 1 (FL)
Helicobacter spp	*Eimeria sp*	Mouse adenovirus type 2 (K87)
Klebsiella pneumoniae/oxytoca	*Encephalitozoon cuniculi*	Mouse cytomegalovirus
Mycoplasma pulmonis	*Entamoeba muris*	Mouse hepatitis virus
Pasteurella spp	*Giardia* muris	Mouse polyomavirus
Proteus mirabilis	*Hymenolepis diminuta*	Mouse rotavirus
Pseudomonas aeruginosa	*Hymenolepis nana*	Mouse thymic virus
Salmonella spp	*Myobia musculi*	Mousepox (*Ectromelia*) virus
Staphylococcus aureus	*Myocoptes musculinus*	Murine norovirus
Streptobacillus moniliformis	Non-pathogenic protozoa (such as *Hexamastix*, *Tritrichomonas*, etc.)	Parvovirus: Minute virus of mice Mouse parvovirus
Streptococci β-hemolytic (not group D)	*Pneumocystis* murina	Pneumonia virus of mice
Streptococcus pneumoniae	*Polyplax species*	Reovirus type 3
	Radfordia spp	Sendai virus
	Spironucleus muris	Theiler's murine encephalomyelitis virus
	Syphacia sp	
	Trichomonas spp	

Supplementary Table 1. Exclusion list of the Plateau Central d'Elevage et d'Archivage, CNRS, Montpellier (SOPF animal facility). Detection tests include RT-PCR, ELISA or Multiplexed Fluorometric ImmunoAssay (Bead).
